# Substituted 2-Aminobenzothiazoles Salicylidenes Synthesis and Characterization as Cyanide Sensors in Aqueous Medium

**DOI:** 10.3390/s18072219

**Published:** 2018-07-10

**Authors:** Anas G. Elsafy, Hala Sultan Al-Easa, Yousef M. Hijji

**Affiliations:** Department of Chemistry and Earth Sciences, Qatar University, Doha 2713, Qatar; Anas.moustafa@qu.edu.qa (A.G.E.); haleasa@qu.edu.qa (H.S.A.-E.)

**Keywords:** 2-aminobenzothiazoles, cyanide, fluorescence, sensors, microwave assisted synthesis

## Abstract

(E)-2-((benzo[*d*]thiazol-2-ylimino)methyl)-4-nitrophenol **1** and (E)-2-(((6-methoxybenzo[*d*]thiazol-2-yl)imino)methyl)-4-nitrophenol **2** were synthesized efficiently under microwave conditions. The structures were confirmed using IR, ^1^H NMR, and ^13^C NMR. UV-vis. Fluorescence investigations demonstrated that **1** and **2** are sensitive and selective sensors for detection of cyanide over all other anions SCN^−^, AcO^−^, N_3_^−^, H_2_PO_4_^−^, H_2_AsO_4_^−^, F^−^, Cl^−^, Br^−^, and I^−^ in aqueous media. Cyanide induces colorimetric change from pale yellow to dark yellow and from transparent to pale yellow for **1** and **2,** respectively. It enhances the absorption at wavelengths 385 nm and 425 nm of **1** and 385 nm and 435 nm of **2**. Acidic anions H_2_PO_4_^−^ and H_2_AsO_4_^−^ displayed significant interference with the interaction of cyanide and sensors **1** and **2**. Sensor **1** has lower detection limit (LDL) 1 × 10^−6^ M, while **2** has LDL 1.35 × 10^−6^ M.

## 1. Introduction

Cyanide detection is very crucial since it might cause death if inhaled or digested. It causes intracellular hypoxia by binding to ferric ion in cytochrome oxidase a_3_ within the mitochondria [1]. Sources of cyanide contamination are wide. Artificial sources of cyanide are numerous such as industrial waste from polyacrylonitrile manufacturing, use in metallurgy, gold mining, and cyanide fishing. Natural sources of cyanide contamination are forest fires and food products such as cassava, seeds of bitter almonds, apples, and apricots in the form of cyanogenic glycosides [2]. The allowed level of cyanide in drinking water according to World Health Organization (WHO) should not exceed 2 µg/L [3].

Anion’s sensing has gained scientists interest because of its importance in a wide variety of applications. Recently, many reports published on the rational design of chemical sensors that are able to detect cyanide selectively at micromolar level based on nucleophilic addition [4]. A chemical sensor should have a binding site that is connected covalently to a signalling unit (chromophore) that responds to the interaction with the anion giving a signal. The signal may be due to the electronic changes in the transition in π → π∗ bonds giving rise to absorption and emission spectral changes. Electronic structure change induced signal transduction takes place upon binding of cyanide to the binding site which is part of conjugated system of the signalling unit [5].

Recently, many molecular probes have been developed as cyanide sensors with various functionalities such as acyltriazene [6], carboxamide [7], squarane [8], trifluoroacetophenone [9], acridinium [10], oxazine [11], cyanine dyes and related inodoliminium NIR dyes [12], and Schiff bases. Schiff bases are widely used as sensors for anions [13,14,15,16,17,18] and cations [19,20,21]. They exhibit excellent sensing abilities due to their electronic structure upon interaction changes with cyanide, solubility in a wide range of solvents, and stability against oxidative and reductive conditions [20].

Different techniques were used to detect cyanide such as voltammetry [22,23], ion chromatography [24], colorimetric [25,26,27,28], and UV-vis and fluorescence [12,17,29,30,31,32,33,34]. UV-vis and fluorescence techniques present many advantages such as immediate easy detection, low cost, high sensitivity and quantification of the anions in different solvent systems including biological systems [35].

Benzothiazole probes are not widely used as cyanide sensors. A previous study [36] reported using benzothiazole derivative as cyanide sensor, but with no interference study. Modification of the structure enhanced the sensitivity of the probes. In this article, we are reporting the synthesis of two substituted 2-aminobenzothiazole salicylidenes under microwave conditions. The sensors were evaluated for their selectivity and sensitivity towards cyanide by UV-vis and fluorescence spectroscopy.

## 2. Experimental

### Reagents and Instruments

2-hydroxy-5-nitrobenzaldehyde, benzo[*d*]thiazol-2-amine, 6-methoxybenzo[*d*]thiazol-2-amine, MeOH, EtOH and inorganic sodium salts of (CN^−^, SCN^−^, AcO^−^, N_3_^−^, H_2_PO_4_^−^, H_2_AsO_4_^−^, F^−^, Cl^−^, Br^−^, and I^−^) were all purchased from sigma-Aldrich (Steinheim, Germany) and dissolved in ultrapure water using Direct-Q 5 UV.

The ^1^H and ^13^C NMR spectra were recorded on a Bruker AVANCE-400 spectrometer (*Bruker* BioSpin, Billerica, MA) operating at 400 and 101 MHz respectively using DMSO-d_6_ as a solvent. IR spectra were obtained using PerkinElmer FT-IR spectrometer (PerkinElmer, Waltham, MA, USA). UV-vis spectra were carried out using Agilent 8453 machine (Agilent, Santa Clara, CA, USA) in 1.0 cm quartz cuvette. Fluorescence peaks were carried out on PerkinElmer LS 45 Fluorescence spectrometer using 1.0 cm quartz cuvette. Scan speed was 700 nm min^−1^, excitation slit was 10 nm, and emission slit was 10 nm. The microwave reactions were carried out in a biotage initiator 8 instrument (Biotage, Uppsala, Sweden). The temperature and time were pre-set as required. The pressure was monitored and indicated.

Sensor **1**: (E)-2-((benzo[*d*]thiazol-2-ylimino)methyl)-4-nitrophenol was dissolved in CH_3_CN as stock solution (1 × 10^−2^ M). Stock solutions of sodium salts of all anions were made in ultrapure water (1 × 10^−1^ M). UV-vis measurements were carried out using sensor (5 × 10^−5^ M) against anions (1 × 10^−4^ M) in H_2_O/CH_3_CN 90:10. Fluorescence measurements were carried out using sensor (1 × 10^−4^ M) against anions (1 × 10^−3^ M) in H_2_O/CH_3_CN 90:10 as final concentrations and excitation wavelength was set at 350 nm.

Sensor **2**: (E)-2-(((6-methoxybenzo[*d*]thiazol-2-yl)imino)methyl)-4-nitrophenol was dissolved in CH_3_CN as stock solution. UV-vis measurements were carried out using sensor (5 × 10^−5^ M) against anions (1 × 10^−4^ M) in H_2_O/CH_3_CN 90:10. Fluorescence measurements were carried out using sensor (5 × 10^−5^ M) against anions (1 × 10^−4^ M) in H_2_O/CH_3_CN 90:10 and excitation wavelength was set at 400 nm.

## 3. Synthesis

### 3.1. Synthesis of ***1***

benzo[*d*]thiazol-2-amine (0.631 g, 4.2 mmol) and 2-hydroxy-5-nitrobenzaldehyde (0.668 g, 4 mmol) were stirred together at room temperature in methanol (5 mL). The mixture was microwaved at 150 °C for 5 min under pressure of 5 bars to give immediate orange precipitate. Crude orange product was filtered and washed twice by cold methanol then recrystallized from ethanol to afford **1** (E)-2-((benzo[*d*]thiazol-2-ylimino)methyl)-4-nitrophenol (0.997 g, 83%) as orange solid; m.p. 224–226 °C; elemental analysis results were; C, 55.9%; H, 3.01%; N, 13.98%. Calc. for C_14_H_9_N_3_O_3_S; C, 56.1%; H, 3.03%; N, 14.04%. IR (ν_max_/cm^−1^); OH (*br*, 2360–3500), C=N (1690), C-N (1490); ^1^H NMR (400 MHz, DMSO-*d*_6_) δ ppm 6.99–7.03 (m, 1H), 7.18–7.23 (m, 2H), 7.32–7.34 (m, 1H), 7.64–7.66 (dd, *J* = 7.83, 0.73 Hz, 1H), 8.34–8.37 (dd, *J* = 9.05, 2.93 Hz, 1H), 8.43–8.44 (d, *J* = 2.93 Hz, 1H), 10.31 (s, 1H), 11.58 (*br*, s, 1H); ^13^C NMR (101 MHz, DMSO-*d*_6_) δ ppm 118.15, 119.01, 121.30, 121.34, 122.70, 124.93, 125.90, 131.20, 131.31, 140.20, 153.13, 166.25, 166.91, 189.59.

### 3.2. Synthesis of ***2***

6-methoxybenzo[*d*]thiazol-2-amine (0.757 g, 4.2 mmol) and 2-hydroxy-5-nitrobenzaldehyde (0.668 g, 4 mmol) were stirred together at room temperature and dissolved in methanol (5 mL). The mixture was microwaved at 150 °C for 5 min under pressure of 5 bars to give immediate orange precipitate. The crude orange product was filtered and washed twice by cold methanol then recrystallized from ethanol to afford **2** (E)-2-(((6-methoxybenzo[*d*]thiazol-2-yl)imino)methyl)-4-nitrophenol (0.9604 g, 73%) as orange solid; m.p. 256–258 °C. Elemental analysis results were; C, 54.54%; H, 3.3%; N, 12.3%. Calc. for C_15_H_11_N_3_O_4_S; C, 54.7%; H, 3.37%; N, 12.7%, IR (ν_max_/cm^−1^); OH (*br*, 2500–3250), C=N (1680), C-N (1495); ^1^H NMR (400 MHz, DMSO-*d*_6_) δ ppm 3.74 (s, 3H) 6.80–6.83 (dd, *J* = 8.80, 2.69 Hz, 1H) 7.18–7.25 (m, 3H) 8.35–8.38 (dd, *J* = 9.16, 3.05 Hz, 1H) 8.44–8.45 (d, *J* = 2.93 Hz, 1H) 10.31 (s, 1H) 12.25 (*br*, s, 1H); ^13^C NMR (101 MHz, DMSO-*d*_6_) δ ppm 56.02, 106.04, 113.38, 118.50, 119.04, 122.70, 124.95, 131.18, 132.29, 140.14, 147.09, 154.79, 165.25, 166.33, 189.62.

## 4. Results and Discussion

Sensors **1** and **2** were synthesized efficiently under microwave conditions in 5 min compared to the long conventional reflux processes that took 1 h [4] or 2 h [36]. Their chemical structures were confirmed using IR, ^1^H NMR, and ^13^C NMR. The presence of the p-nitro group increases the aldehyde reactivity that will facilitate the condensation reaction. The imines were obtained in good yields.

**Figure 1:**
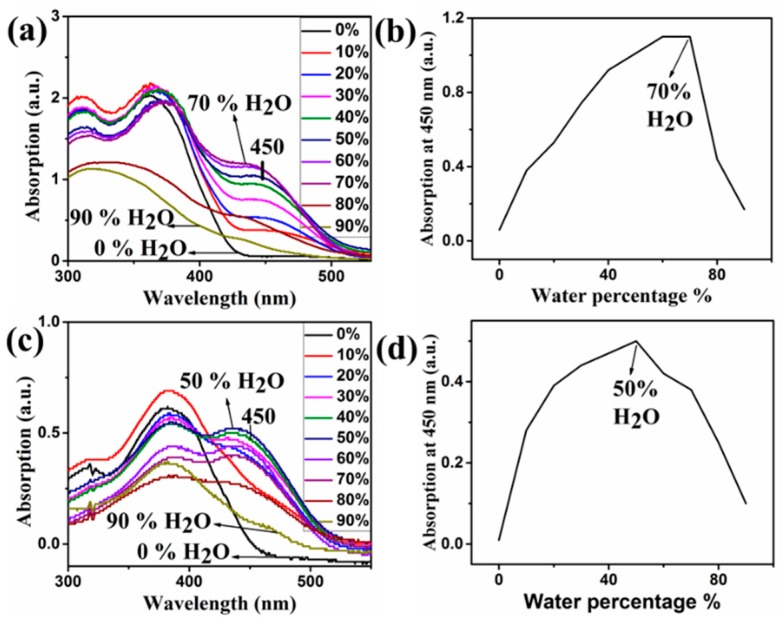
(**a**,**b**) UV-vis spectra of the absorbance of **1** and plot of absorbance measured as a function of water concentration at 450 nm; (**c**,**d**) UV-vis spectra and absorbance of **2** measured at 450 nm as a function of water percent in graphical representation.

Absorption behavior of **1** and **2** is greatly influenced by the solvent. The solvent effect was investigated by increasing the polarity of the solvent. In CH_3_CN, sensor **1** has an absorption peak at 360 nm, while sensor **2** has an absorption peak at 385 nm. Escalating water ratio from 0% to 90% displays that absorption of both sensors in acetonitrile is affected upon addition of water. Figure 1a,b show that increasing water ratio resulted in increased absorption of the new peak of **1** at wavelength 450 nm until it reaches the maximum absorption at 70% water. Increasing the water ratio beyond 70% resulted in absorption decrease. Such reduction in the absorption may be due to the reduction in the solubility of the imine in the mixed solvents. Figure 1c,d show that absorption change of the peak at 450 nm of **1** and **2**. First, the peak is increased upon increasing water ratio to 50%, where dissociation increases with polar protic solvent increase. Further addition of water led to absorption decrease due to the decrease in the solubility.

**Scheme 1:**
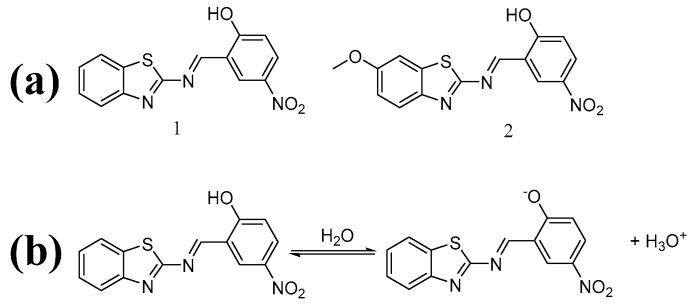
(**a**) Chemical structure of **1** and **2**; (**b**) Equilibrium forms of **1** in polar solvents.

Sensors **1** and **2** exist in equilibrium between protonated and deprotonated forms in polar solvents, Scheme 1b. As water ratio increases in the solvent, the solubility of the sensors is enhanced and equilibrium is directed towards the deprotonated form. Sensor **2** is less soluble in polar solvents than **1** due to the presence of methoxy group. As deprotonation increases upon escalating the ratio of water, the absorption of the new peak at 450 nm increases until it reaches the maximum absorption at 70% for **1** and 50% water for **2**. Further increase of water ratio lowered the solubility of the sensors and directed the equilibrium towards the protonated form resulting in absorption decrease.

**Figure 2:**
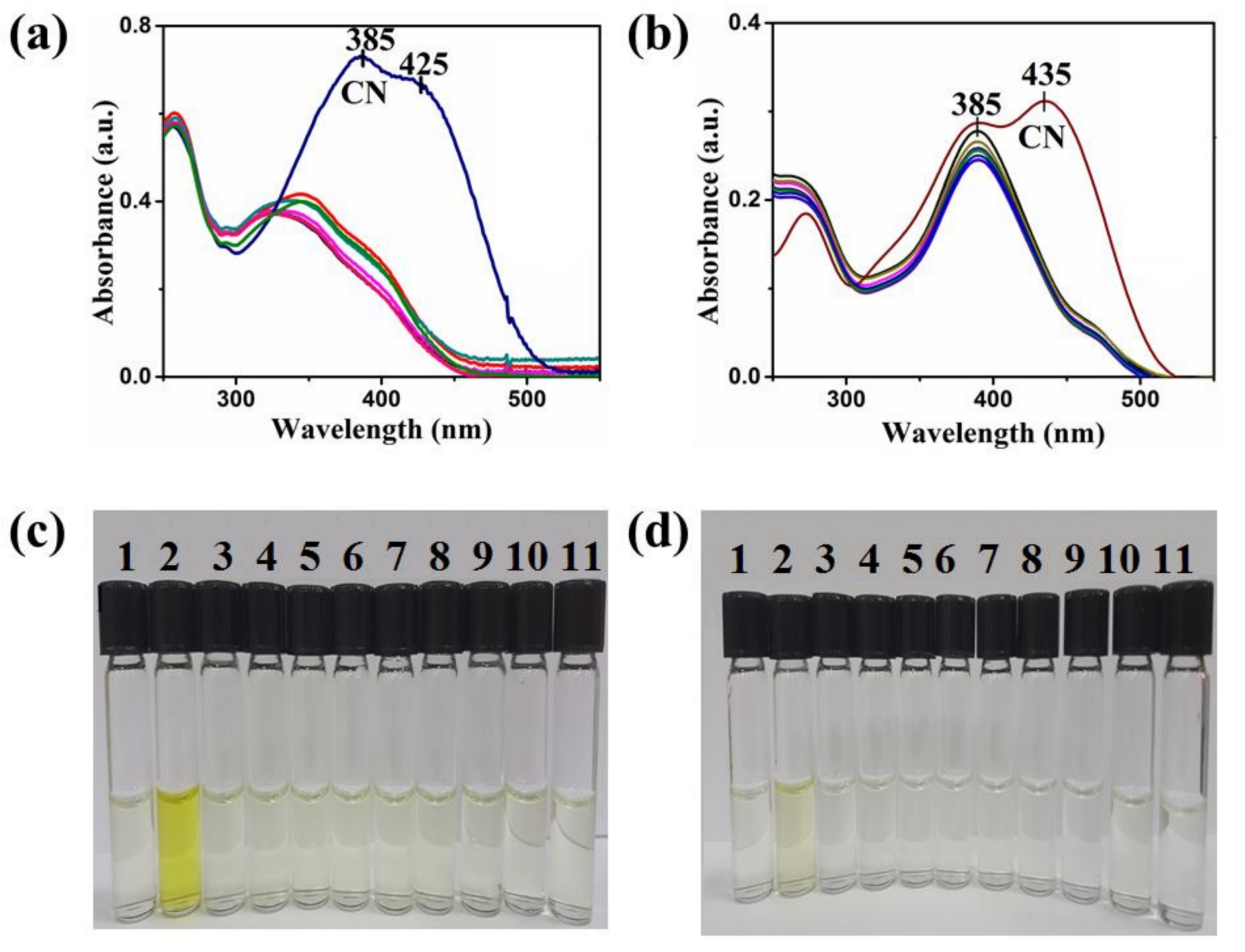
(**a**) UV-vis Spectra of **1** in the presence of different anions n water: acetonitrile 90:10: (**–1**; **–** SCN^−^, **–**N_3_^−^, **–**I^−^, **–**H_2_PO_4_^−^, **–**H_2_AsO_4_^−^, **–**F^−^, **–**CN^−^, **–**Cl^−^, **–**+Br^−^, **–**AcO^−^), (**b**) UV-vis Spectra of **2** in the presence of different anions (**– 2**; **–** SCN^−^, **–**N_3_^−^, **–**I^−^, **–**H_2_PO_4_^−^, **–**H_2_AsO_4_^−^, **–**F^−^, **–**CN^−^, **–**Cl^−^, **–**Br^−^, **–**AcO^−^). (**c**) and (**d**) color change of **1** and **2** in the presence of various anions respectively: (1: **1**; 2: CN^−^; 3: AcO^−^; 4: N_3_^−^; 5: H_2_PO_4_^−^; 6: H_2_AsO_4_^−^; 7: SCN^−^; 8: F^−^; 9: Cl^−^; 10: Br^−^; 11: I^−^).

Sensors **1** and **2** are evaluated for their sensitivity and selectivity to different anions using mono basic inorganic sodium salts of the anions CN^−^, SCN^−^, AcO^−^, N_3_^−^, H_2_PO_4_^−^, H_2_AsO_4_^−^, F^−^, Cl^−^, Br^−^, and I^−^ in H_2_O/CH_3_CN 90:10 visually, UV-vis and Fluorescence spectroscopy. Both sensors showed selectivity towards CN^−^ over all other anions, as seen in Figure 2a,b.

Sensors **1** and **2** have absorption peaks at 325 nm and 385 nm, respectively. Cyanide enhanced the absorption intensity at 385 nm and 425 nm of sensor **1**, whereas it enhanced the absorption intensity at 385 nm and 435 nm of **2**. Other anions resulted in no significant absorption intensity change. Figure 2b,c display visual color change of **1** from pale yellow to dark yellow and **2** from transparent to pale yellow upon addition of CN^−^.

**Figure 3:**
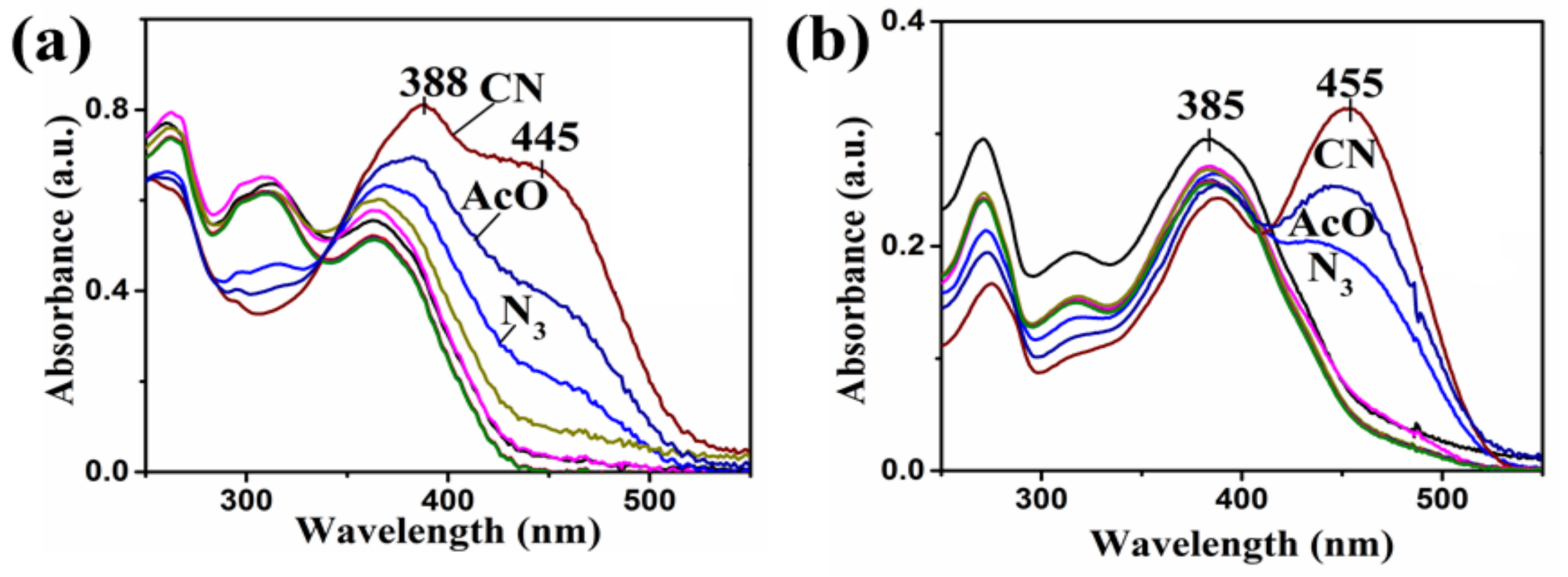
(**a**) UV-vis Spectra of **1** in the presence of different anions in water: acetonitrile 10:90 (**–1**; **–**SCN^−^, **–**N_3_^−^, **–**I^−^, **–**H_2_PO_4_^−^, **–**H_2_AsO_4_^−^, **–**F^−^, **–**CN^−^, **–**Cl^−^, **–**+Br^−^, **–**AcO^−^), (**b**) UV-vis Spectra of **2** in the presence of different anions in water: acetonitrile 10:90 (**– 2**; **–** SCN^−^, **–**N_3_^−^, **–**I^−^, **–**H_2_PO_4_^−^, **–**H_2_AsO_4_^−^, **–**F^−^, **–**CN^−^, **–**Cl^−^, **–**Br^−^, **–**AcO^−^).

Altering the solvent ratio from H_2_O/CH_3_CN from 90:10 to become 10:90 changed the previous results. Basic anions such as AcO^−^, N_3_^−^ enhanced absorption intensity at 388 nm and 445 nm of **1** and 455 nm of **2**, Figure 3. These results indicated that sensors **1** and **2** lose their selectivity for cyanide in less polar solvents.

Selectivity of the sensors towards different anions is based on the nature of the anions. Cyanide is more nucleophilic than other reported anions and has less hydrogen bonding ability [36], so that the imine carbon of **1** and **2** is more susceptible to cyanide addition. Other anions have larger solvation effect with protic solvents than cyanide [17] and form hydrogen bonds in H_2_O/CH_3_CN 90:10. Sensors **1** and **2** have the electron-withdrawing nitro group in *p-*position rendering the phenolic group very acidic and liable for deprotonation by basic anions such as AcO^−^ and N_3_^−^ in less polar solvent H_2_O/CH_3_CN 10:90.

**Scheme 2:**
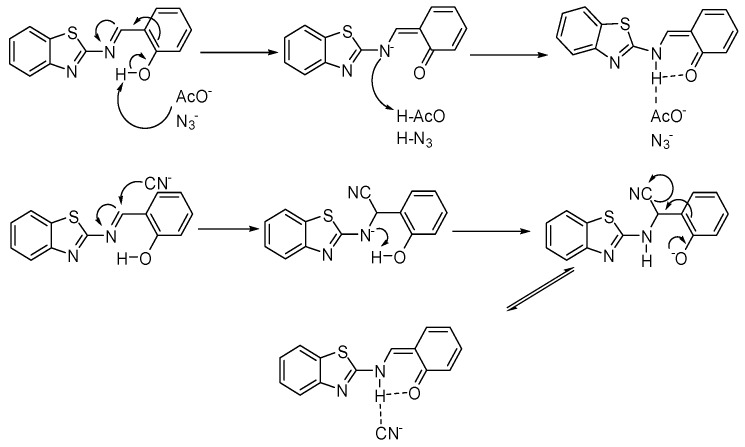
Proposed mechanism of the interaction of **1** and **2** with different anions.

The proposed mechanism of the interaction of **1** and **2** with anions was previously reported [36]. Intramolecular proton transfer takes place resulting in the keto-amine form via two pathways; (i) addition of cyanide to the electron-deficient carbon atom of the imine group and (ii) deprotonation of phenolic hydrogen by basic anions, Scheme 2.

Sensors **1** and **2** have λ_max_ < 400 nm. The absorption at λ_max_ > 400 nm is enhanced upon addition of cyanide. The λ_max_ < 400 nm is attributed to the enol form and the λ_max_ > 400 nm is attributed to the keto-amine form [37,38]. Scheme 2 displays the conversion of phenol-imine form into keto-amine form with intramolecular proton transfer.

**Figure 4:**
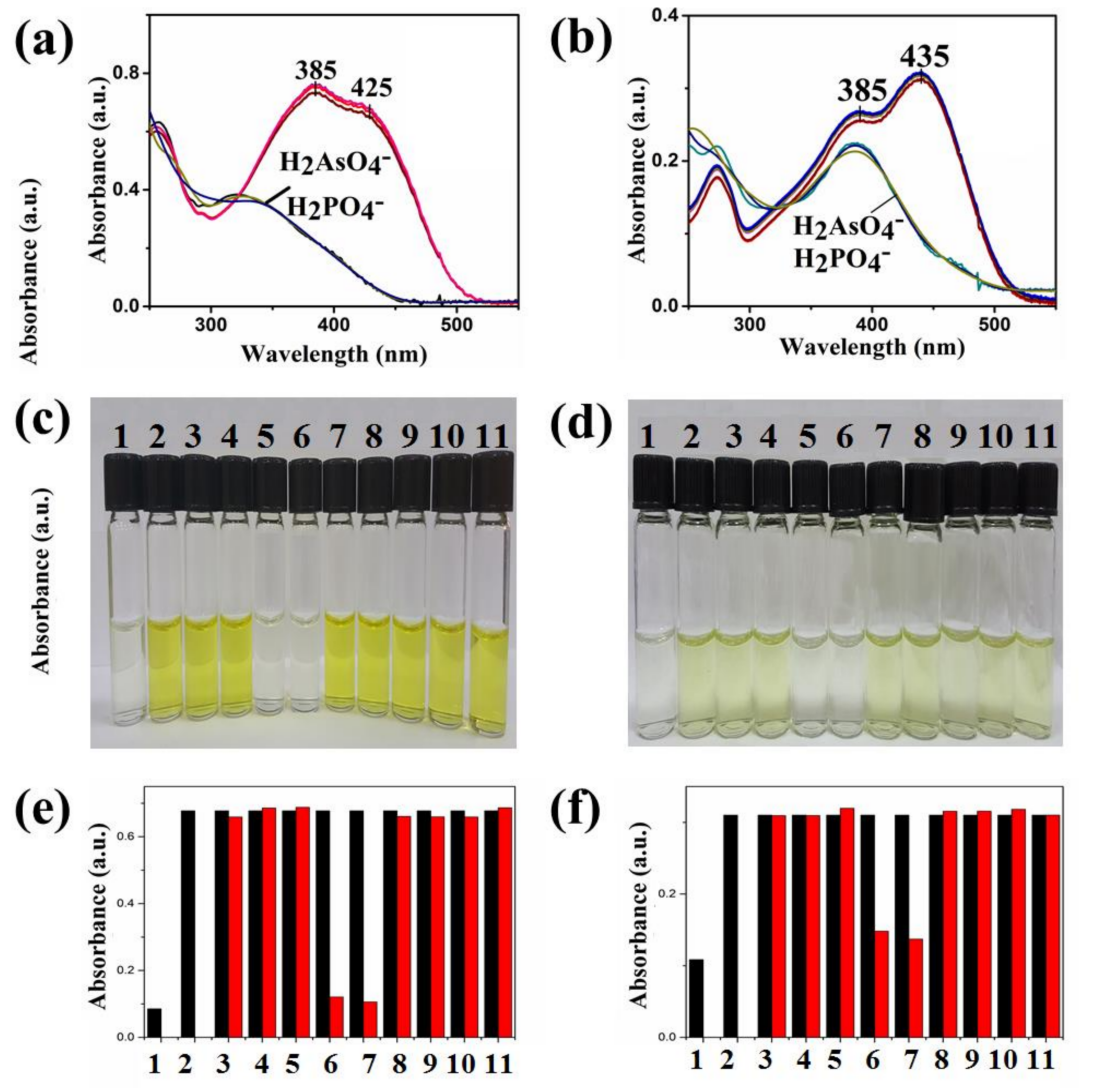
(**a**) and (**b**) UV-vis spectra interference of **1** and **2 **in the presence of cyanide with different anions respectively: interfering anions (**–**CN^−^, **–**N_3_^−^, **–**I^−^, **–**H_2_PO_4_^−^, **–**H_2_AsO_4_^−^, **–**F^−^, **–**SCN^−^, **–**Cl^−^, **–**Br^−^, **–**AcO^−^), (**c**) and (**d**) colorimetric change of **1** and **2** in the presence of cyanide and interfering anions: (1: sensor; 2: CN^−^; 3: AcO^−^; 4: N_3_^−^; 5: H_2_PO_4_^−^; 6: H_2_AsO_4_^−^; 7: SCN^−^; 8: F^−^; 9: Cl^−^; 10: Br^−^; 11: I^−^). (**e**) and (**f**) bar grams of absorbance change of **1** and **2** in the presence of cyanide and interfering anions respectively (1: sensor; 2: CN^−^; 3: AcO^−^; 4: N_3_^−^; 5: H_2_PO_4_^−^; 6: H_2_AsO_4_^−^; 7: SCN^−^; 8: F^−^; 9: Cl^−^; 10: Br^−^; 11: I^−^).

Investigation of reported anions interference with CN^−^ was carried out by addition of the reported anions 10^−3^ M to the mixture of the sensors 5 × 10^−5^ M and cyanide 10^−4^ M. Major quenching effect on the reactivity of **1** and **2** towards CN^−^ by acidic anions H_2_AsO_4_^−^ and H_2_PO_4_^−^ was observed, Figure 4a,b. Figure 4c,d display visual color change upon addition of H_2_AsO_4_^−^ and H_2_PO_4_^−^ to the mixtures of **1** and **2** with CN^−^. Bar graphs of the interference effect of the reported anions on the reactivity of **1** and **2** towards cyanide are shown in (Figure 4e,f).

Sensors **1** and **2** showed intensity reduction at the λ_max_ 385 nm and 425 nm of **1** and 385 nm and 435 nm of **2**. This reduction is referred to protonation of both CN^−^ and deprotonated form of the sensors by acidic anions H_2_AsO_4_^−^ and H_2_PO_4_^−^ directing the equilibrium towards the protonated form of the sensors.

**Figure 5:**
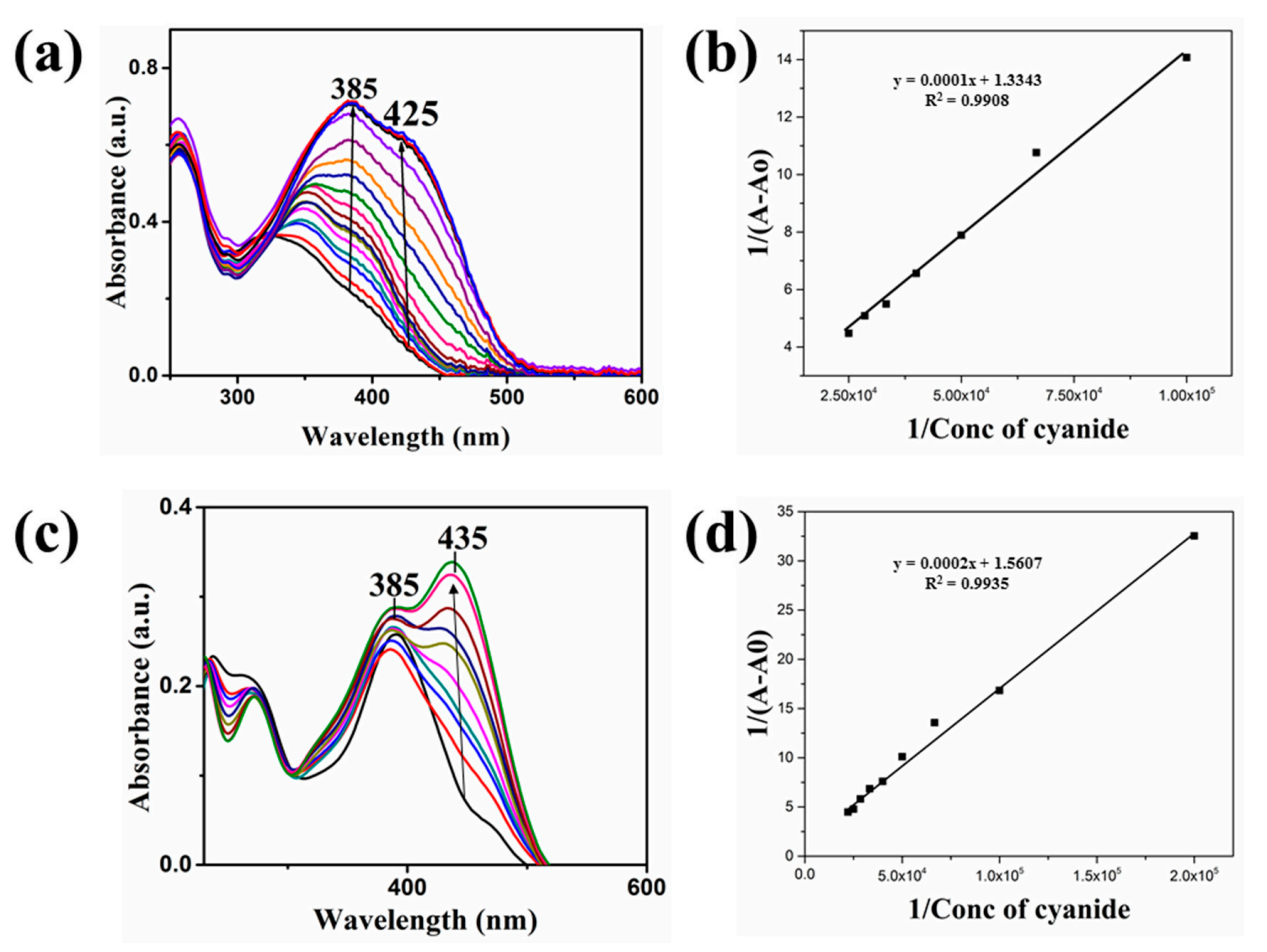
(**a**,**c**) Sensors **1** and **2** titrations with cyanide from 5 × 10^−6^ M to 8 × 10^−5^ M and from 5 × 10^−6^ to 5 × 10^−5^ M, respectively; (**b**,**d**) Benesi–Hildebrand plots of the reaction of **1** and **2** respectively with cyanide.

Titration of sensors **1** and **2** with varying cyanide concentrations showed direct increase in the absorption at 385 nm and 425 nm of **1** and 385 nm and 435 nm of **2**, Figure 5a,c with cyanide concentration. Benesi–Hildebrand plots are presented for each sensor (Figure 5b,d), from which Ka values were calculated. Moreover, lower detection limits (LDL) and isosbestic points are shown in Table 1.

**Figure 6:**
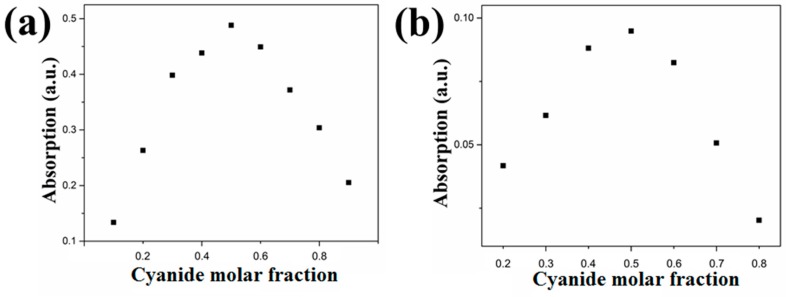
Job plots of **1** (**a**) and **2** (**b**). The monitored wavelengths are 425 nm and 435 nm, respectively.

Job plots of **1** and **2** displayed in Figure 6 indicating 1:1 complex from the sensors with CN^−^. The calculated LDL of **1** is much lower than the allowed level of cyanide in drinking water stated by World Health Organization (WHO), which should not exceed 2 µg/L [3].

**Figure 7:**
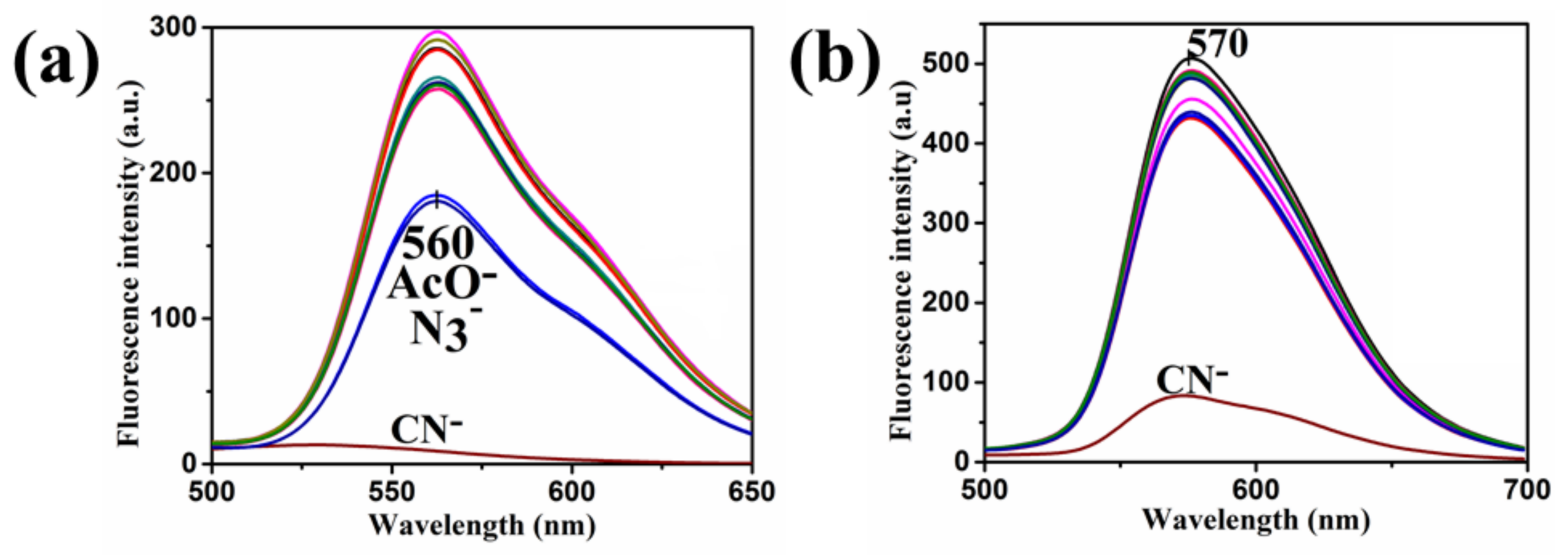
(**a**) and (**b**) fluorescence spectra of **1** and **2** in the presence of different anions: (**–** SCN^−^, **–**N_3_^−^, **–**I^−^, **–**H_2_PO_4_^−^, **–**H_2_AsO_4_^−^, **–**F^−^, **–**CN^−^, **–**Cl^−^, **–**Br^−^, **–**AcO^−^).

Investigation of the fluorescence behavior of **1** and **2** was carried out to evaluate their selectivity and sensitivity towards cyanide. Fluorescence results greatly confirmed the UV-vis results. Addition of reported anions to **1** and **2** displayed selectivity of these sensors towards CN^−^, Figure 7. Sensors **1** and **2** witnessed fluorescence intensity reduction selectively after addition of CN^−^. Sensor **1** displayed partial fluorescence intensity reduction in presence of basic anions such as AcO^−^ and N_3_^−^.

Intramolecular charge transfer (ICT) plays a very important role in the fluorescence behaviors of **1** and **2** since reduced ICT results in fluorescence intensity enhancement, whereas efficient ICT quenches the fluorescence intensity [39]. Addition of cyanide to the electron deficient imine carbon of the sensors resulted in the formation of the keto form which induced the ICT throughout the molecules leading to fluorescence intensity reduction of **1** and **2**. The presence of the electron-withdrawing, nitro group in **1** enhanced the acidity of the phenolic group enabling deprotonation by basic anions AcO^−^ and N_3_^−^.

**Figure 8:**
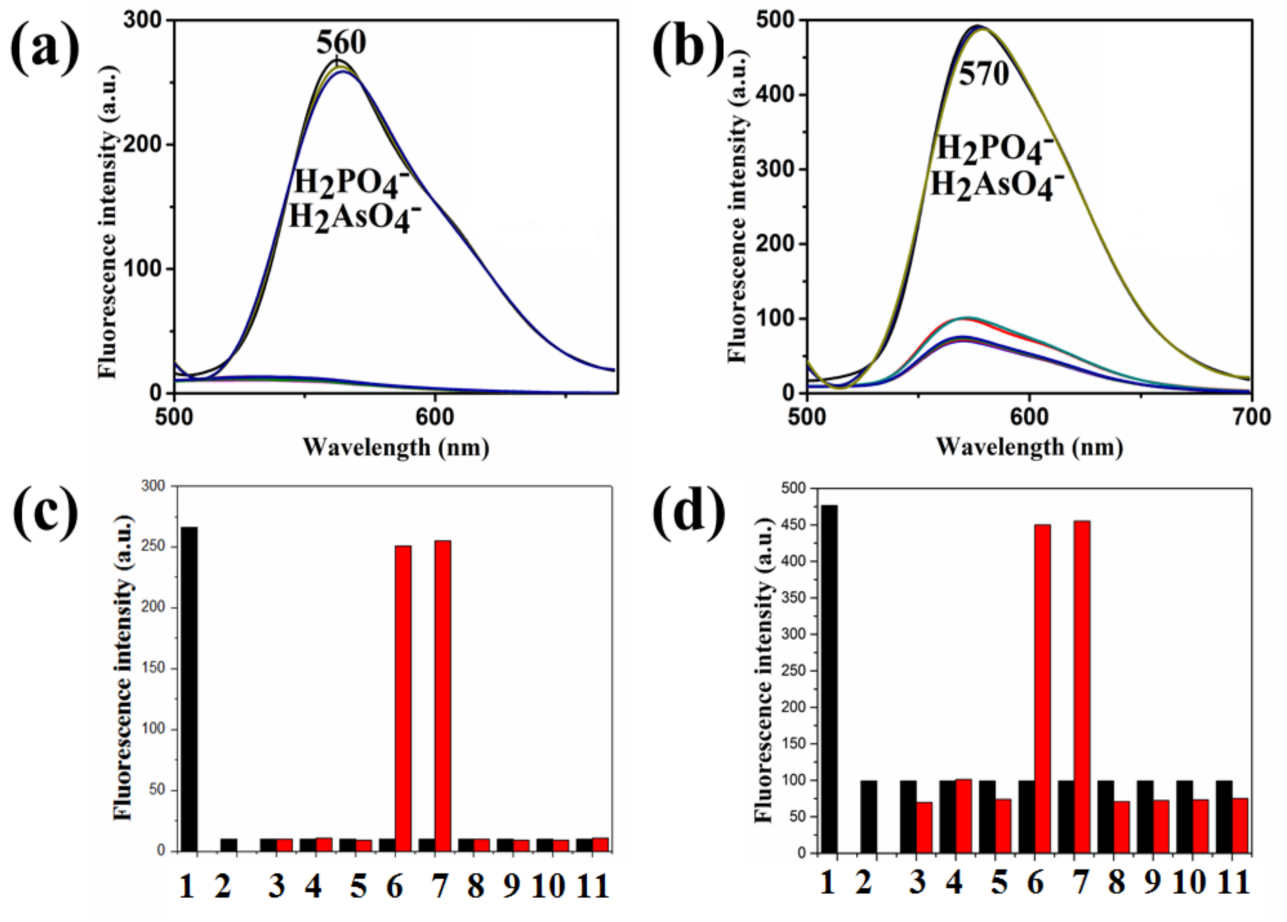
(**a**) and (**b**) Fluorescence spectra of **1 **and **2** respectively in the presence of cyanide and interfering anions.: (**– 1**, **–1** + CN^−^, **–**N_3_^−^, **–**I^−^, **–**H_2_PO4^−^, **–**H_2_AsO_4_^−^, **–**F^−^, **–**SCN^−^, **–**Cl^−^, **–**Br^−^, **–**AcO^−^), (**c**) and (**d**) bar grams fluorescence intensity change of **1** and **2** in the presence of cyanide and different anions.(1: **2**; 2: **2** + CN^−^; 3: SCN^−^; 4: N_3_^−^; 5: AcO^−^; 6: H_2_AsO_4_^−^; 7: H_2_PO_4_^−^; 8: F^−^; 9: Cl^−^; 10: Br^−^; 11: I^−^).

Interference of H_2_PO_4_^−^ and H_2_AsO_4_^−^ with sensors **1** and **2** reactivity towards CN^−^ is shown in Figure 8a,b. Remarkably, H_2_PO_4_^−^ and H_2_AsO_4_^−^ quenched the sensors reactivity towards cyanide. Formation of the protonated form of the sensors inhibited the ICT, thus re-induced the fluorescence intensity. Bar graphs of the interference of reported anions with sensors **1** and **2** reactivity towards cyanide are well displayed in Figure 8c,d.

## 5. Conclusions

Sensors **1** and **2** were synthesized efficiently and evaluated for their interaction with a wide range of anions. They showed selectivity towards cyanide in H_2_O/CH_3_CN 90:10 while all other reported anions were solvated in water. Acidic anions such as H_2_AsO_4_^−^ and H_2_PO_4_^−^ quenched the interaction of the sensors towards cyanide. LDL of the sensors is lower than the allowed level in water recommended by WHO.

## Figures and Tables

**Table 1:** Ka values, lower detection limits and isosbestic points of **1** and **2**.

Compound	K_a_ (M^−1^)	LDL (M)	Isosbestic Point
**1**	13,343	1 × 10^−6^	325 nm
**2**	7803	1.35 × 10^−6^	305 nm
